# The Role of Renewable Resources for Ecology and Human Health

**DOI:** 10.3390/life13040879

**Published:** 2023-03-25

**Authors:** Milka Mileva

**Affiliations:** Department of Virology, The Stephan Angeloff Institute of Microbiology, Bulgarian Academy of Sciences, 26, Acad. Georgi Bonchev Str., 1113 Sofia, Bulgaria; milkamileva@gmail.com

Scientists are increasingly asking very serious and topical questions: what do we throw away as waste from industrial production? Are there valuable ingredients in what we throw away? What are we not using up from the waste from agriculture or the food industry? What are the environmental pollutants? Are there useful practices and models for converting this waste?

These questions gave us the idea to create the Special Issue “The Role of Renewable Resources for Ecology and Human Health”.

Day by day, many natural resources that were abundantly available to humans are diminishing and becoming scarce. Factors such as poor agricultural practices, logging, mining and mineral exploration, industrial and technological development, overconsumption, and waste generation, etc., contribute to this. This leads to environmental pollution and the depletion of natural resources.

Colleagues from different fields of science have shown interest in these issues. We accepted and printed 10 articles with very interesting scientific facts. The pruning wastes (leaves and stems) of rose farms in Taif are rich in cardiac glycosides and flavonoids and inhibit the growth *of Bacillus subtilis*, *Escherichia coli*, *Proteus vulgaris*, *Aspergillus fumigatus*, and *Candida albicans* [1]. Steam distillation of valuable rose essential oil from Bulgarian *Rosa damascena* Mill. and *Rosa alba* L. generates large volumes of wastewater. Although such wastewaters are biopollutants, they contain valuable bioactive compounds that have cytoprotective/genoprotective effects and can reduce DNA damage [2].

An experiment conducted to evaluate the yield and economic benefits of a rice-wheat cropping system (RWCS) influenced by the co-application of sewage sludge (SSL) and fertilizer shows that partial replacement of chemical fertilizers with organic fertilizers reduces both environmental pollution and economic costs and provides greater soil health benefits [3].

Kabaivanova et al. (2022) adapted a fast, efficient process for biogas/biomethane production from natural wheat straw and corn stover. The biomethane yield in biogas reached up to 60% between the second and fifth day [4].

Many plant sources are used as well in cooking as spices and food additives, and so they are involved indirectly in human metabolism through the daily food intake manifesting their healing effect. While extracts from the leaves of *Ginkgo biloba* L. are widely studied and relatively well-known and used in the pharmaceutical industry, the therapeutic properties of the seeds are less well-studied. Petrov et al. (2022) looked for beneficial biological properties of a methanolic extract of *Ginkgo biloba* seeds. The good ability of the extract to neutralize free radicals could have a beneficial effect in pathological conditions with an etiology of oxidative stress [5].

In retrospect, herbal medicinal plants have been used for thousands of years as an alternative healing remedy, including for the treatment of various viral diseases. Natural products used to treat viral infections have some advantages over the chemotherapeutics used. Products derived from medicinal plants are more easily absorbed by the body due to their natural origin and exhibit fewer side effects as they are structurally similar to normal cellular components. The last three years have shown humanity a significant example of the dangers and seriousness of the prevention, treatment, and control of viral diseases through the COVID-19 pandemic. The search for new approaches to control the pathogenesis of this viral infection with extremely diverse symptoms is the basis of a study conducted by Vilhelmova-Ilieva et al. on testing the antiviral activity of a group of Bulgarian medicinal plants widely used to alleviate the symptoms characteristic of COVID-19 patients. The extracts of 13 Bulgarian medicinal plants showed excellent redox-modulating potential and could find application as antioxidants and antiviral symptomatic treatment agents in the fight against COVID-19 infection [6].

The preventive effect of another natural product—snail mucus *Cornu aspersum* (Müller, 1774)—has been investigated as an antiulcerogenic agent on the pathogenesis of ethanol-induced gastric ulcer [7]. The protection of mucus in this model of gastric damage is probably due to the complex action of many factors, including the protection of molecules with antioxidant properties, substances that provide tissue regeneration, and compounds with antimicrobial properties effects that may contribute to the beneficial effect of snail mucus in stomach ulcers and make it a suitable substance for the prevention of stomach diseases.

The complex of classical cytogenetic analyses on the cytotoxic and genotoxic activity of the hydrosols obtained from the water-vapor distillation of Bulgarian *Rosa alba* L. and *Rosa damascena* Mill. did not show significant cytotoxic and genotoxic effects [8]. With their fine aroma and low genotoxic risk, they will continue to be a desirable product in various areas of human life.

Global warming and anthropogenic pollution of natural watersheds, including arsenic, is a serious risk to human health. Omeroglu et al. [9] investigated the contamination of the world’s largest Soda Lake—Lake Van, with arsenic and arsenic-resistant bacteria. The authors demonstrate that the total amount of arsenic in this water body changes seasonally and is a serial human health hazard due to the use of lake water for irrigation in agricultural areas. Identification of resistant strains of microorganisms from high-contamination environments, as well as determination of the pathways by which microbial remediation of arsenic occurs, are essential.

In the production process, when processing corn, large amounts of by-products are inevitably generated, which are rich in valuable proteins. Of utmost importance is the development of biotechnologies for the valorization of such by-products as corn-milling by-products from cereals to food/feed ingredients. The selection of optimal conditions under which to preserve the nutritional value of the corn raw material and the total content of amino acids. Thermo-mechanical processing (extrusion), with subsequent 48-h fermentation, is a promising technology that produces a food material capable of potentially reducing microbial contamination, improving its nutritional value, and improving the technological properties of albumins and globulins of corn-milling by-products, as well as the digestibility and bioactivity of prolamins [10].

All these studies show that the source of valuable molecules is often products that are considered waste in technological pollution, or agricultural by-products that are discarded without being recovered. Thus, many of these natural functional ingredients still remain untapped and a clear understanding of the mechanisms of their beneficial properties needs innovative and original research. The innovative and unconventional approaches applied by the authors who published in the first volume of the Special Issue “The Role of Renewable Resources for Ecology and Human Health” have attracted the attention of leading specialists in these fields, and most of the articles now have citations. Therefore, the Editorial Board of Life Journal has suggested that we continue to collect such valuable expertise in Volume II of this edition. My heartfelt thanks to all the authors who responded to our idea and sent us their contributions, to all of you and to the future authors who will publish with us I wish you creative success in your future research, and I look forward with particular interest to your new proposals.

## Data Availability

https://www.mdpi.com/journal/life/special_issues/renewable_resources_life.
